# Fasciae of the musculoskeletal system: normal anatomy and MR patterns of involvement in autoimmune diseases

**DOI:** 10.1007/s13244-018-0650-1

**Published:** 2018-08-29

**Authors:** Thomas Kirchgesner, Xavier Demondion, Maria Stoenoiu, Patrick Durez, Adrien Nzeusseu Toukap, Frédéric Houssiau, Christine Galant, Souad Acid, Frédéric Lecouvet, Jacques Malghem, Bruno Vande Berg

**Affiliations:** 1 Department of Radiology - Musculoskeletal Imaging Unit, Cliniques universitaires Saint-Luc / Institut de Recherche Expérimentale et Clinique (IREC), Université Catholique de Louvain, Brussels, Belgium; 2 Department of Radiology and Musculoskeletal Imaging, CHRU Lille / Laboratory of Anatomy, Faculty of Medicine of Lille, Lille, France; 3 Department of Rheumatology, Cliniques universitaires Saint-Luc, Brussels, Belgium; 4 Department of Pathology, Cliniques universitaires Saint-Luc, Brussels, Belgium

**Keywords:** Fascia, Musculoskeletal, Anatomy, Autoimmune, MRI

## Abstract

**Abstract:**

The fascial system is a three-dimensional continuum of connective tissues present everywhere throughout the body, from the head to the toes and from the skin to the bone. The current article aims to review the normal anatomy of the fasciae of the musculoskeletal system with macroscopic and microscopic correlations and to describe their appearance at MRI in normal subjects and in patients with autoimmune diseases of the musculoskeletal system.

**Key Points:**

• *The fascial system is a three-dimensional continuum of connective tissues.*

• *It is present everywhere throughout the body, from the head to the toes and from the skin to the bone.*

• *The normal fascial system is barely visible at MRI.*

• *MR patterns of fascial involvement in autoimmune diseases reflect the complex anatomy of the fasciae of the musculoskeletal system.*

## Introduction

The fascial system has received little attention in the literature as it has been regarded as fibrous membranes barely involved by abnormal conditions [1]. Developments in the understanding of the functional anatomy of the fasciae have led to a unified anatomical concept of a fascial system present everywhere throughout the body, from the head to the toes and from the skin to the bone [2]. The current article aims to review the normal anatomy of the fasciae of the musculoskeletal system with macroscopic and microscopic correlations and to describe their appearance at MRI in normal subjects and in patients with autoimmune diseases of the musculoskeletal system.

## Definition of the fascial system

Fasciae were defined as “*masses of connective tissue large enough to be visible to the unaided eye*” according to the Grey’s Anatomy textbook [3]. This historical concept has evolved depending on the variable importance attributed to the gross or microscopic anatomy, to the histological composition and to the functions of the fascial system. According to a most recent definition [2], *“the fascial system consists of the three-dimensional continuum of soft, collagen-containing, loose and dense fibrous connective tissues that permeate the body. It incorporates elements such as adipose tissue, adventitiae and neurovascular sheaths, aponeuroses, deep and superficial fasciae, epineurium, joint capsules, ligaments, membranes, meninges, myofascial expansions, periostea, retinacula, septa, tendons, visceral fasciae, and all the intramuscular and intermuscular connective tissues including endo-/peri-/epimysium. The fascial system interpenetrates and surrounds all organs, muscles, bones and nerve fibers, endowing the body with a functional structure, and providing an environment that enables all body systems to operate in an integrated manner”.* This holistic approach emphasizes the links between all membranous components of the skeleton from the skin to the periosteum and from the body to the limb extremities.

## Anatomy

**Fig. 1:**
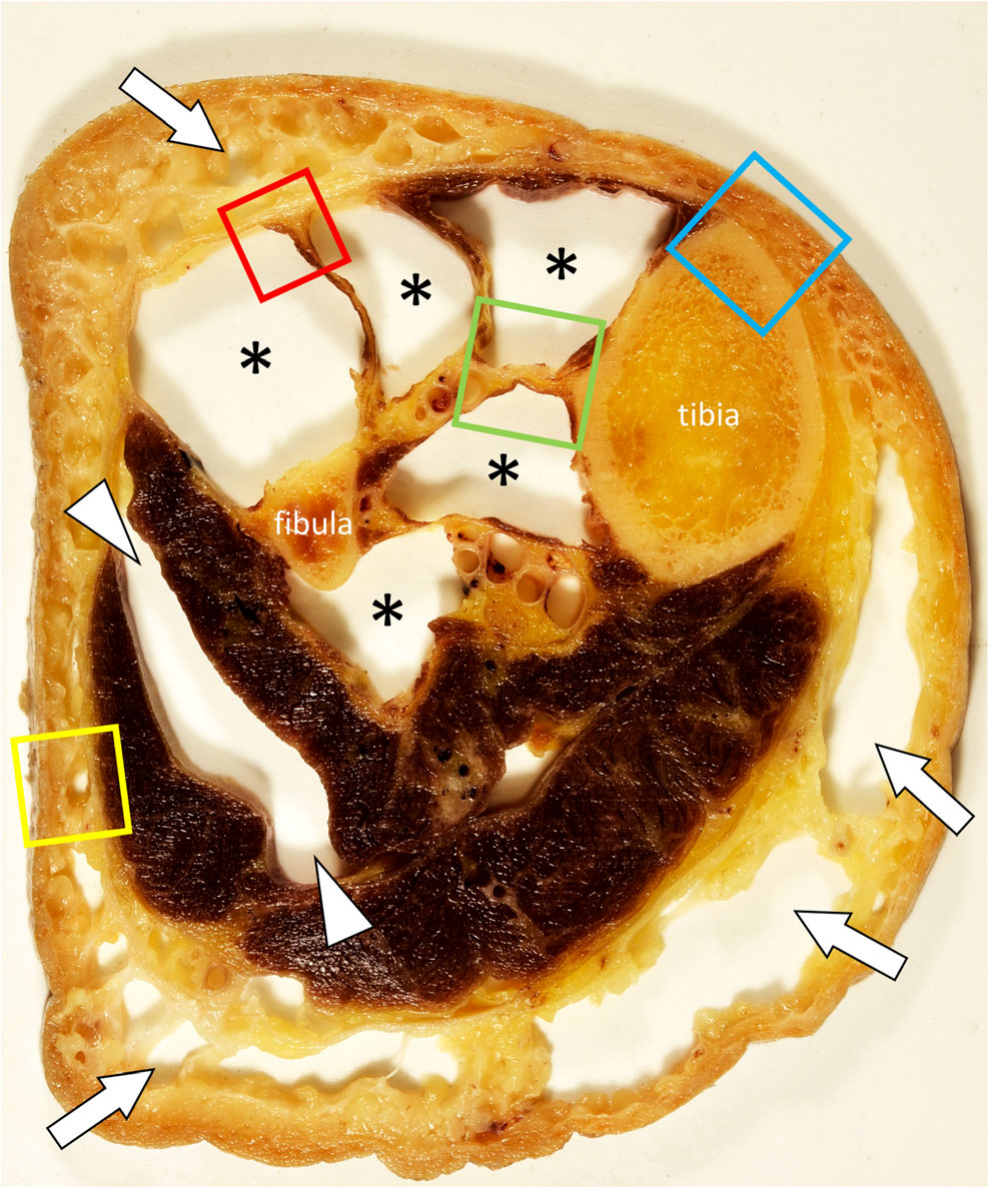
Photograph of a transverse section of a cadaver leg after removal of the subcutaneous fat (arrows) and the muscles of the anterior and deep posterior compartments (asterisks). Muscles of the lateral compartment were separated (arrowheads). Colored squares indicate areas that correspond to Figs. 3, 4, 5 and 6

In the current review, anatomical dissection of a cadaveric leg with histopathological correlation of selected specimens will be used to illustrate the anatomy of the fascial system (Fig. 1). Dissections were performed on a body that had been donated for science research to the Laboratory of Anatomy of Lille, France, in accordance with the ethical standards and French law. All histological images used in this review are stained with Masson’s trichrome stain, which stains connective tissue (collagen) blue.

**Fig. 2:**
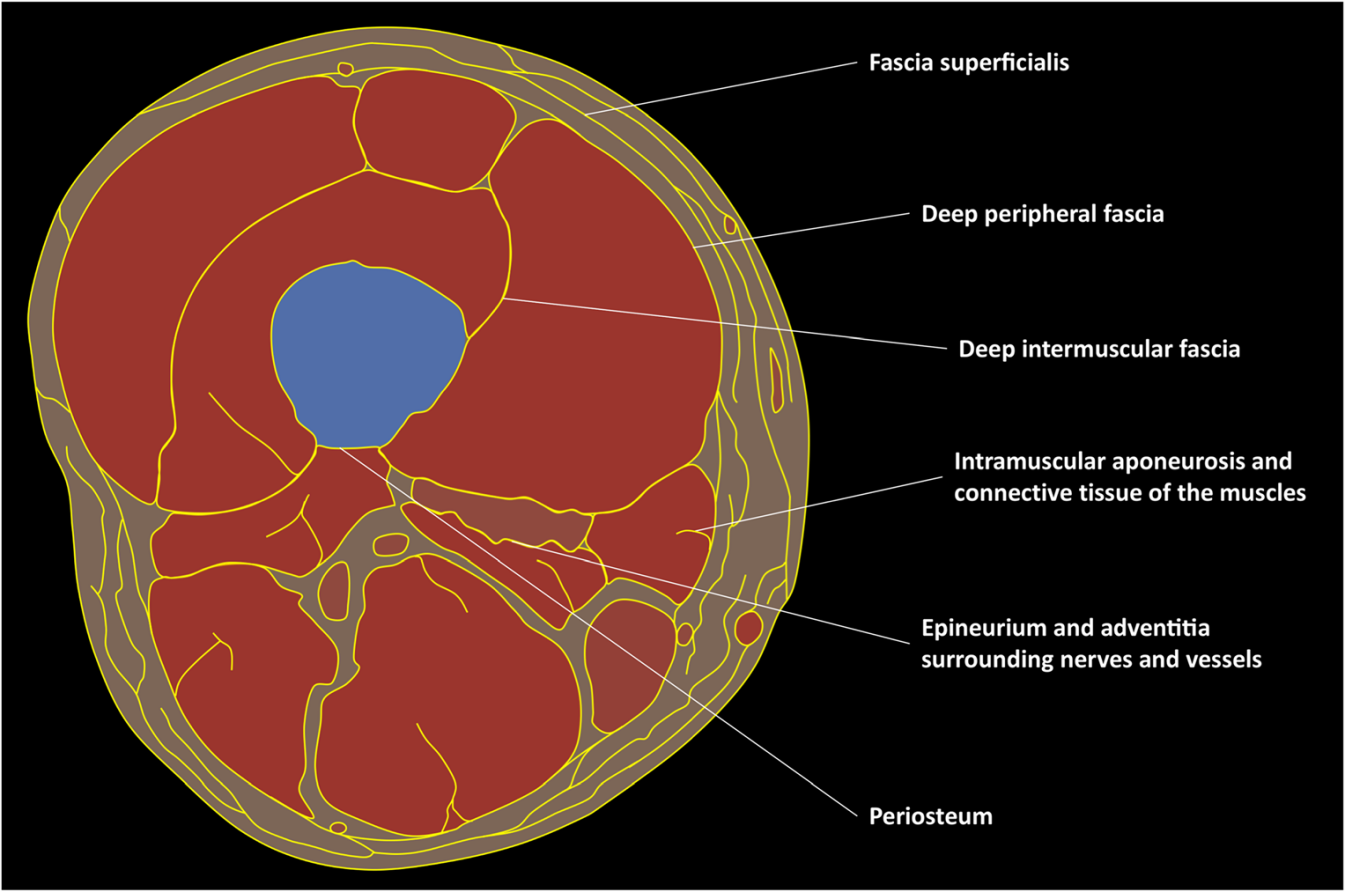
Schematic drawing of a transverse section of the thigh illustrating its fascial anatomy

Anatomists, surgeons and radiologists tend to use different terms to describe the components of the fascial system (Fig. 2) [4].

**Fig. 3:**
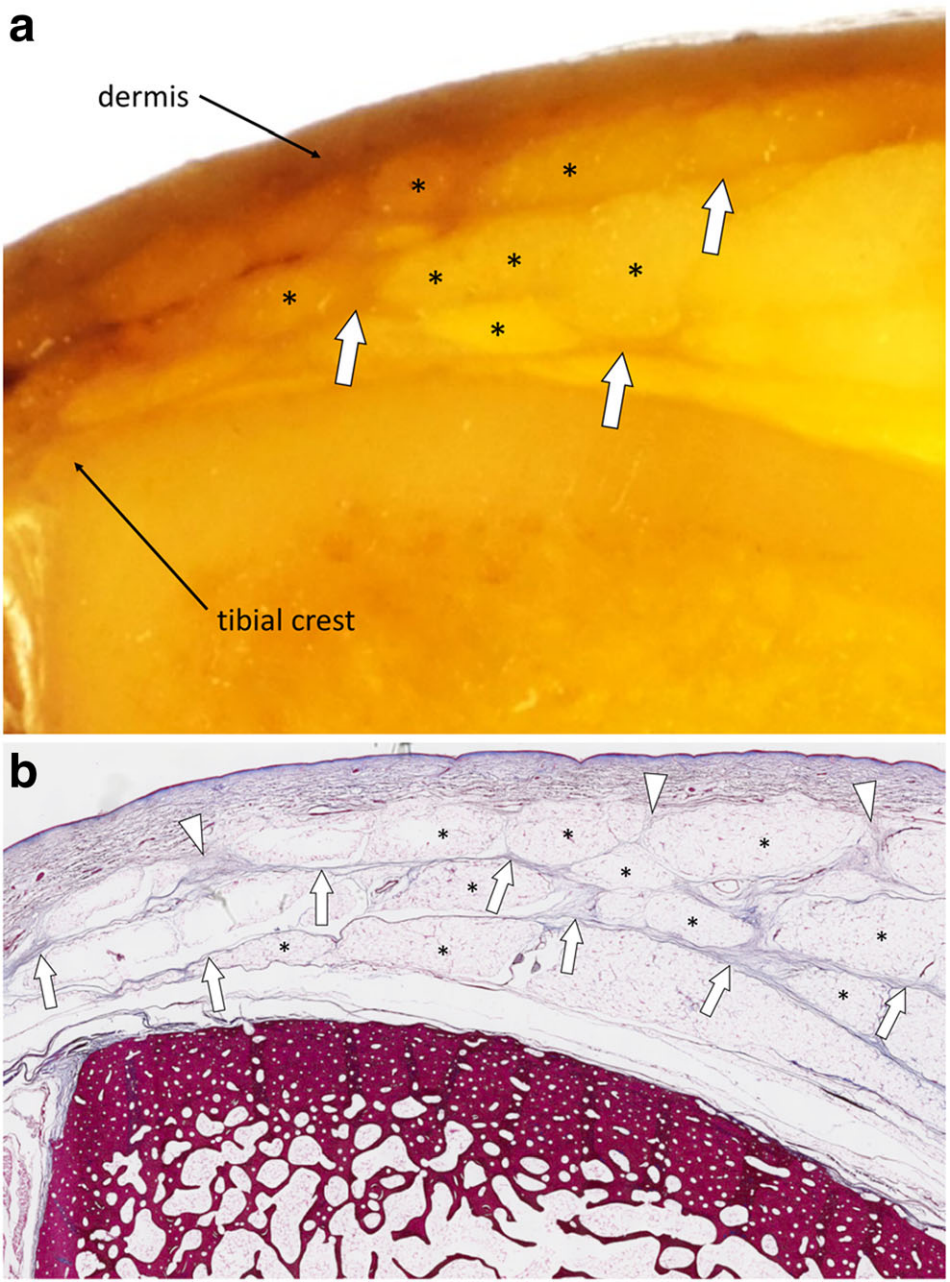
**a** Close-up photograph and (**b**) photograph of the corresponding microscopic image (Masson’s trichrome stain) of the subcutaneous tissues of the antero-medial part of the leg (blue square in Fig. 1). Fat lobules (asterisks) are delimited by connective tissue tracts (arrowheads) anchored to the dermis. These tracts of connective tissue converge and constitute the fascia superficialis (arrows)

The term “fascia superficialis” is used to designate either the entire hypodermis or a layer of connective tissue located immediately deep to the dermis [1, 5]. This layer of connective tissue of variable thickness (*stratum membranosum*) is attached through fibrous tracts to the dermis (*retinacula cutis superficialis*) and to the deeper components of the fascial system (*retinacula cutis profondis)*. It is composed by interwoven collagen fibres, loosely packed and mixed with abundant elastic fibres. Its extent, thickness and topography vary according to the body segment. In this review we use the term “fascia superficialis” to designate the complex formed by this subcutaneous layer of connective tissue and the fibrous tracts that connect it superficially to the dermis and deeply to the underlying deeper components of the fascial system. The so-called fascia superficialis constitutes a three-dimensional connective tissue network in the hypodermis that contains fat lobules and superficial nerves and vessels (Fig. 3).

**Fig. 4:**
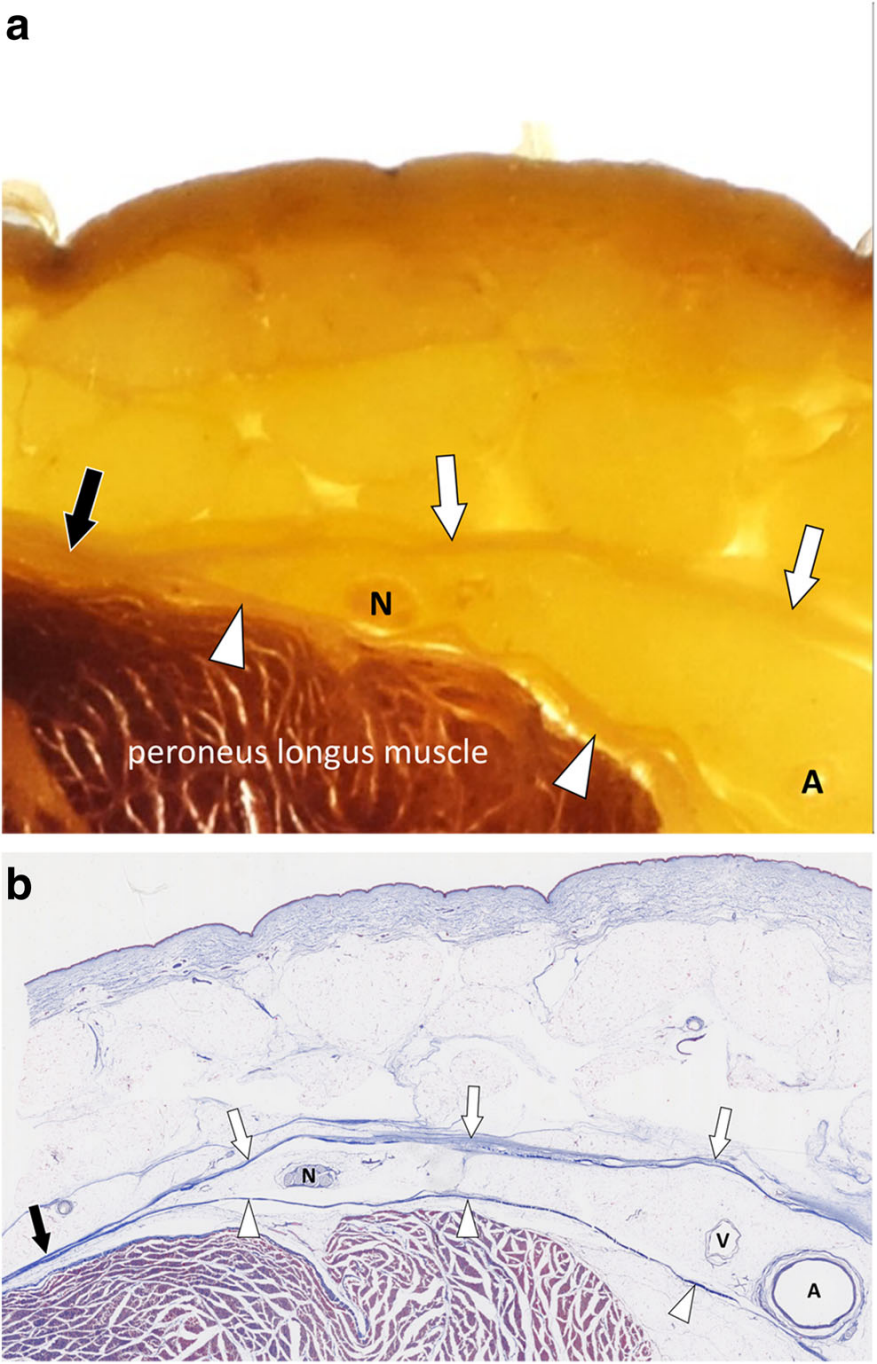
**a** Close-up photograph and (**b**) photograph of the corresponding microscopic image (Masson’s trichrome stain) of the subcutaneous tissues of the lateral part of the leg (yellow square in Fig. 1). The fascia superficialis (white arrows) and the deep peripheral fascia (arrowheads) converge to become macroscopically undistinguishable and microscopically contiguous (black arrows). Superficial nerves (N), arteries (A) and veins (V) run between the fasciae

The term “deep peripheral fascia” designates the layer of connective tissue composed of more tightly packed collagen bundles located at the interface between the hypodermis and connective tissue surrounding the muscles (epimysium). In some areas the fascia superficialis and deep peripheral fascia are contiguous (Fig. 4).

**Fig. 5:**
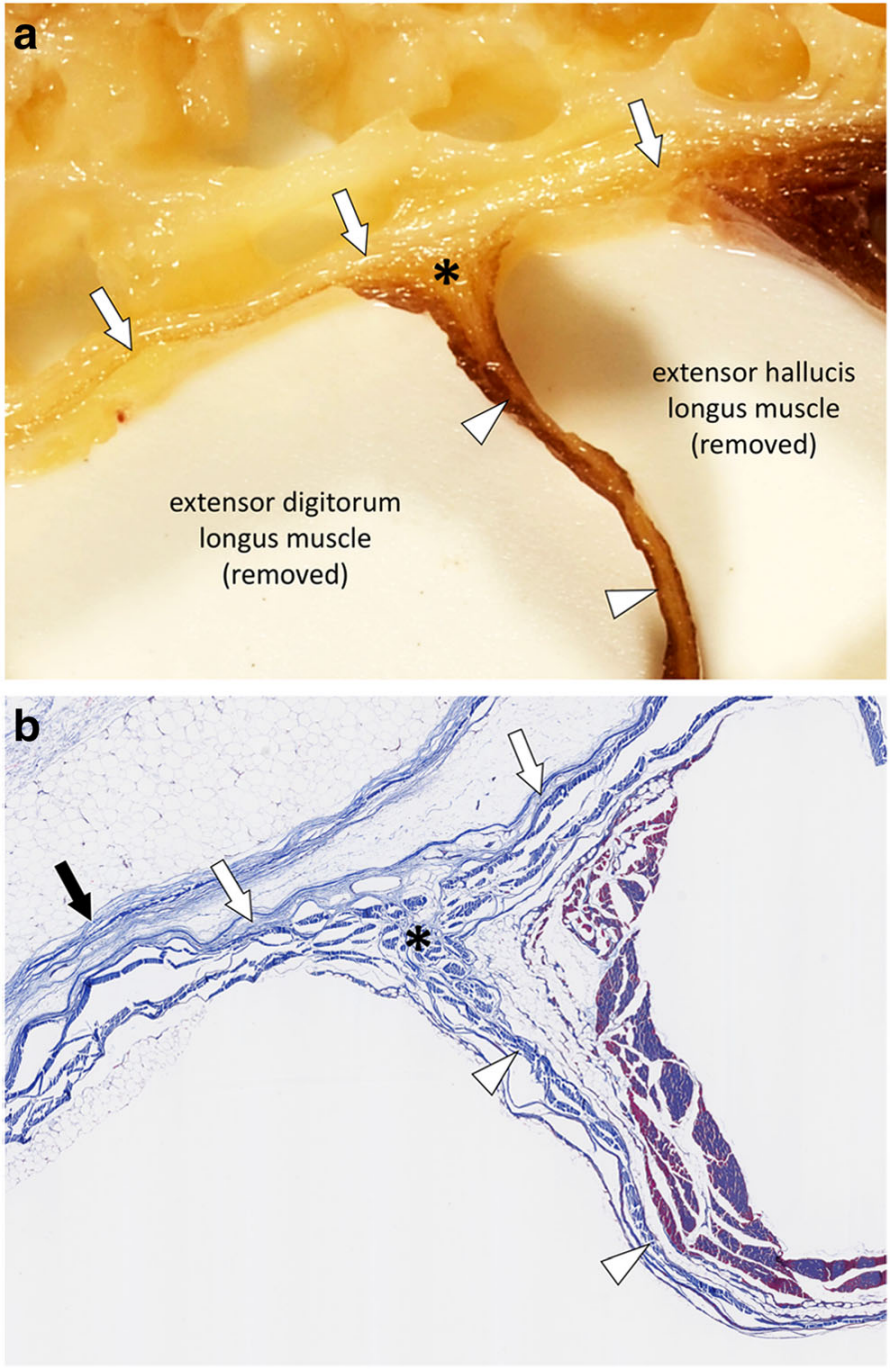
**a** Close-up photograph and (**b**) photograph of the corresponding microscopic image (Masson’s trichrome stain) of the anterior compartment of the leg (red square in Fig. 1). The deep peripheral fascia (white arrows) and the deep intermuscular fascia (arrowheads) are in continuity (asterisks). The fascia superficialis (black arrow) closely overlies the deep peripheral fascia

**Fig. 6:**
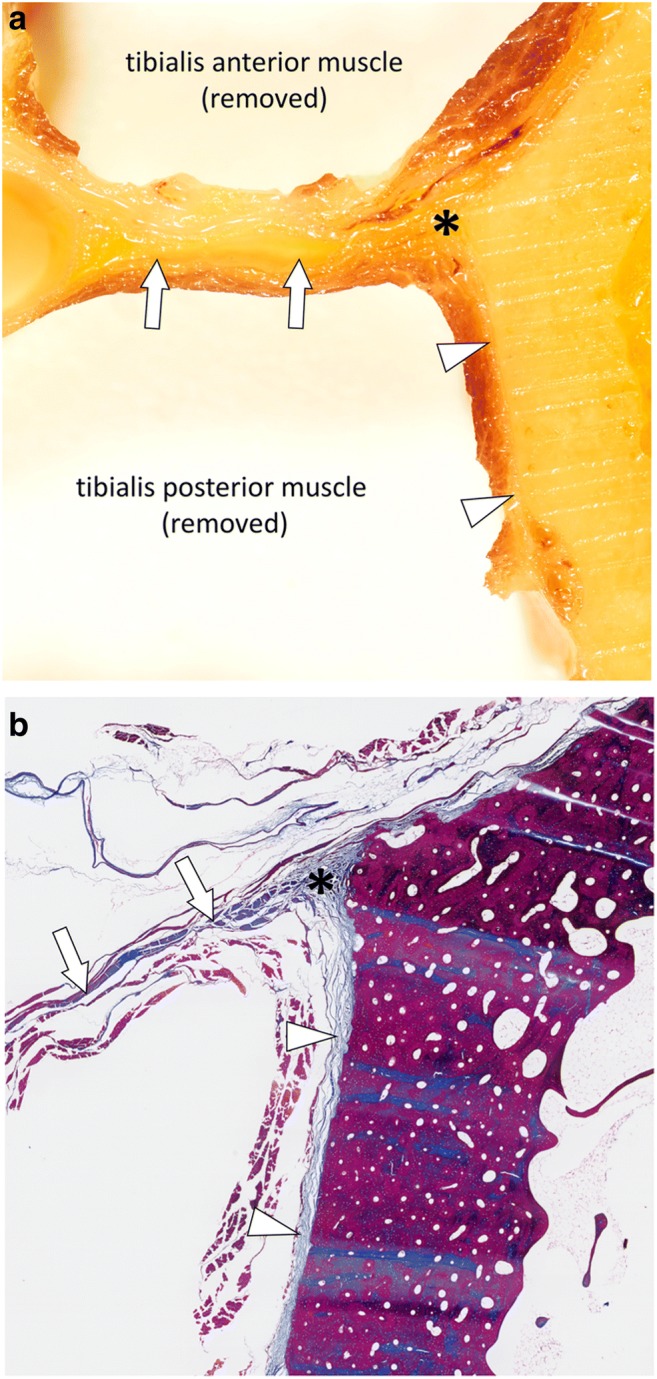
**a** Close-up photograph and (**b**) photograph of the corresponding microscopic image (Masson’s trichrome stain) of the deep posterior compartment and lateral part of the tibia (green square in Fig. 1). The deep intermuscular fasciae (arrows) between the tibialis anterior and tibialis posterior muscles are in continuity (asterisks) with the periosteum of the tibia (arrowheads)

The term “deep intermuscular fascia” is used to describe the intermuscular septa located deep to the deep peripheral fascia and separating muscles and muscle groups from each other (Fig. 5). Deep to the muscles the deep intermuscular fascia is in continuity with the periosteum (Fig. 6).

According to the most recent definition of the fascial system, fasciae also include identifiable anatomical structures (retinacula, interosseous membranes, tendons and entheses) and connective tissues specific to organs like muscles (epimysium and perimysium), nerves (epineurium and perineurium) and vessels (adventitia) [2, 6].

## Normal MR imaging

**Fig. 7:**
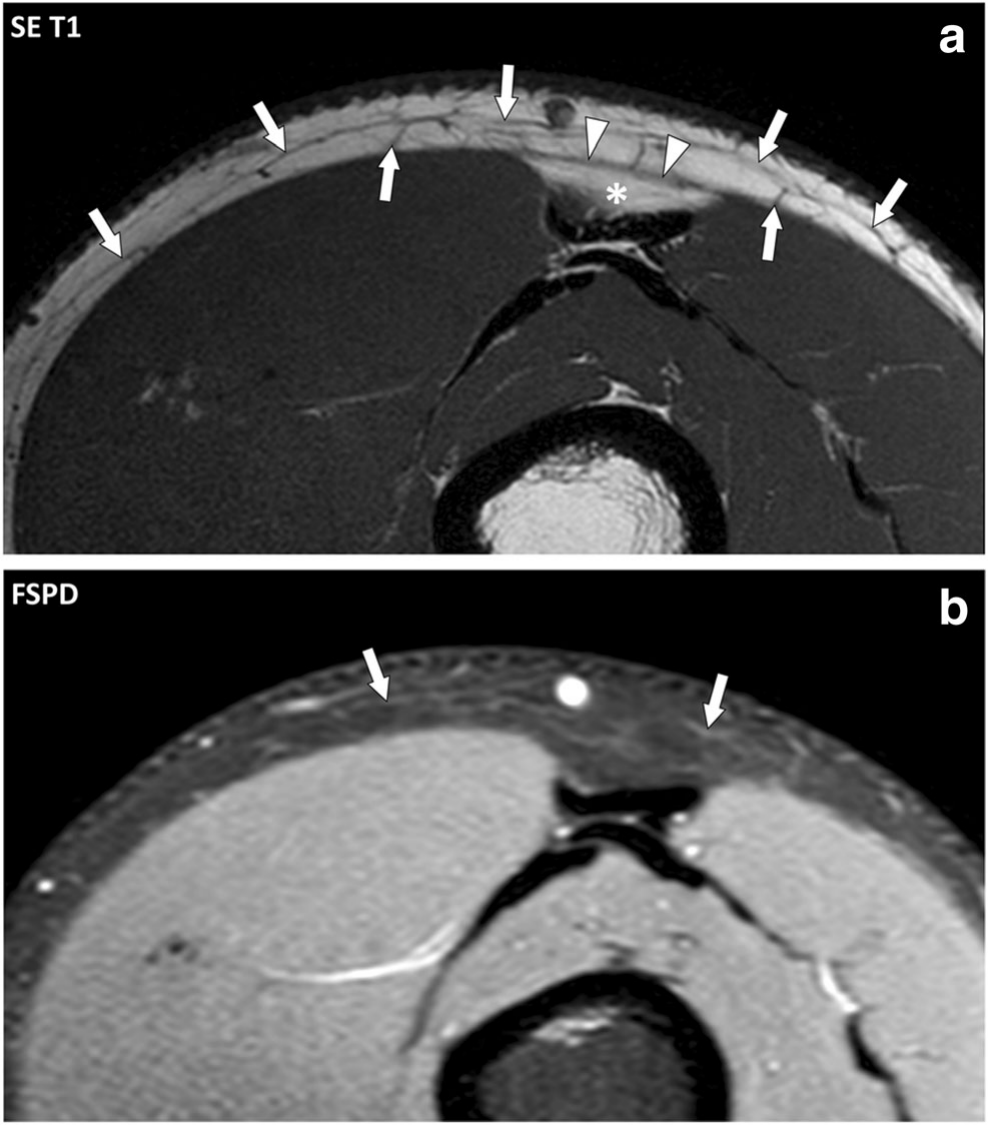
Transverse (**a**) spin echo (SE) T1 and (**b**) fat-suppressed proton density (FSPD)-weighted images of the anterior part of the thigh of a 33-year-old normal subject. On the SE T1-weighted image, the fascia superficialis (arrows) appears as a thin reticular network of low signal intensity embedded in the hypodermic fat. The deep peripheral fascia (arrowheads) is only depicted in areas where fat is present deep to it (asterisk). On the corresponding FSPD-weighted image, the fascial system shows low signal intensity and is almost undistinguishable from the adjacent fat

The normal fascial system is barely visible at MRI because of its anatomical configuration and its high connective tissue content (Fig. 7).

**Fig. 8:**
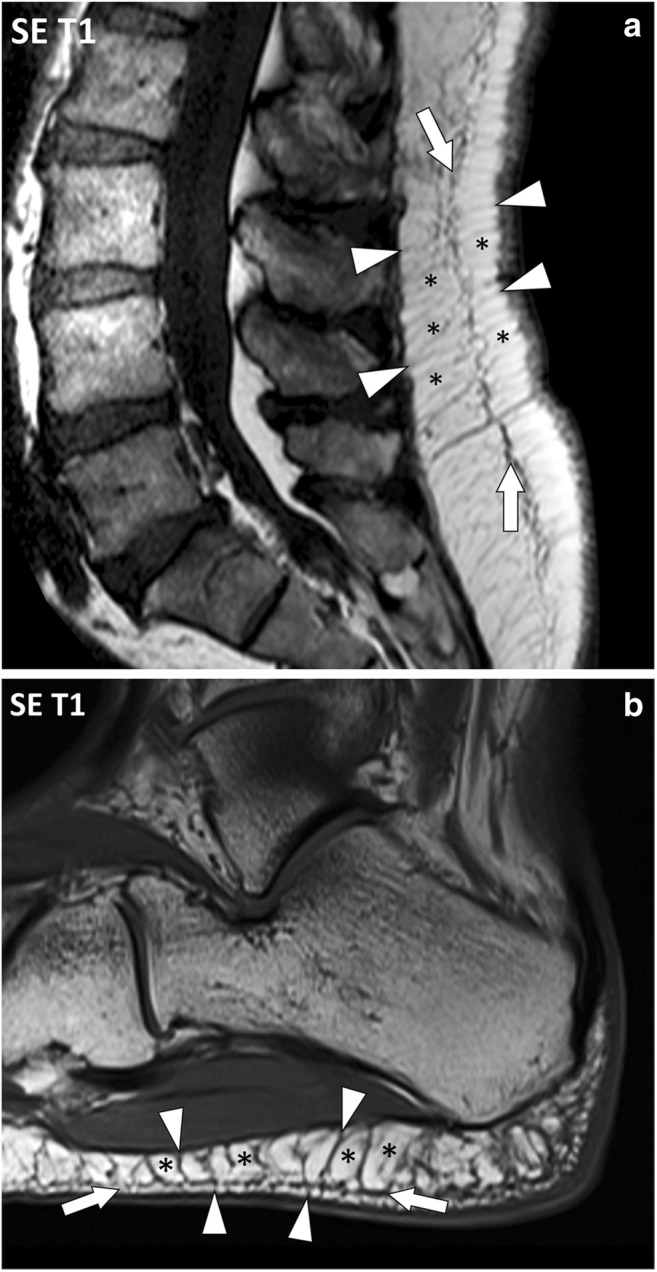
Sagittal SE T1-weighted images of (**a**) the lumbar spine and (**b**) the heel of two different patients. The fascia superficialis (arrows) is well depicted with fibrous tracts (arrowheads) perpendicular to the dermis and to the deep peripheral fascia, delineating fat lobules (asterisks). These attachments vary according to the considered body segment

The fascia superficialis appears as a thin reticular network of low signal intensity on spin echo (SE) T1- and T2-weighted images embedded in the hypodermic fat [7]. It is even less conspicuous on fluid-sensitive fat-suppressed images. Its extent, thickness and topography vary according to the considered body segment (Fig. 8).

The deep peripheral fascia is generally not visible at MRI. When it is surrounded by fat on both sides, it appears as a low signal intensity structure on SE T1- and T2-weighted images located deep to the hypodermis and thicker than the fascia superficialis. Chemical shift artefact may create pseudo-thickening of the normal deep fascia along the frequency encode direction, which should not be interpreted as abnormal.

The deep intermuscular fascia and the periosteum are not visible because of the absence of spontaneous signal contrast between muscles and between muscles and bone.

## MR patterns of involvement in autoimmune diseases

Autoimmune disorders of the musculoskeletal fascial system include numerous diseases such as systemic sclerosis, systemic lupus erythematosus, eosinophilic fasciitis, dermatomyositis, polymyalgia rheumatica and some other mixed connective tissue diseases along with seronegative and seropositive arthritides. In all these conditions, the involvement of the fascial system is highly variable in terms of frequency, selectivity for the components of the fascial system and localization. Each disease tends to target specific components of the fascial system with inconstant involvement of the adjacent structures.

MRI is the most efficient imaging technique to assess inflammatory changes of the fascial system. The following non-exhaustive overview of MR patterns of fascial involvement will be organized according to the site of involvement and not according to each specific disease.

### Fascia superficialis

**Fig. 9:**
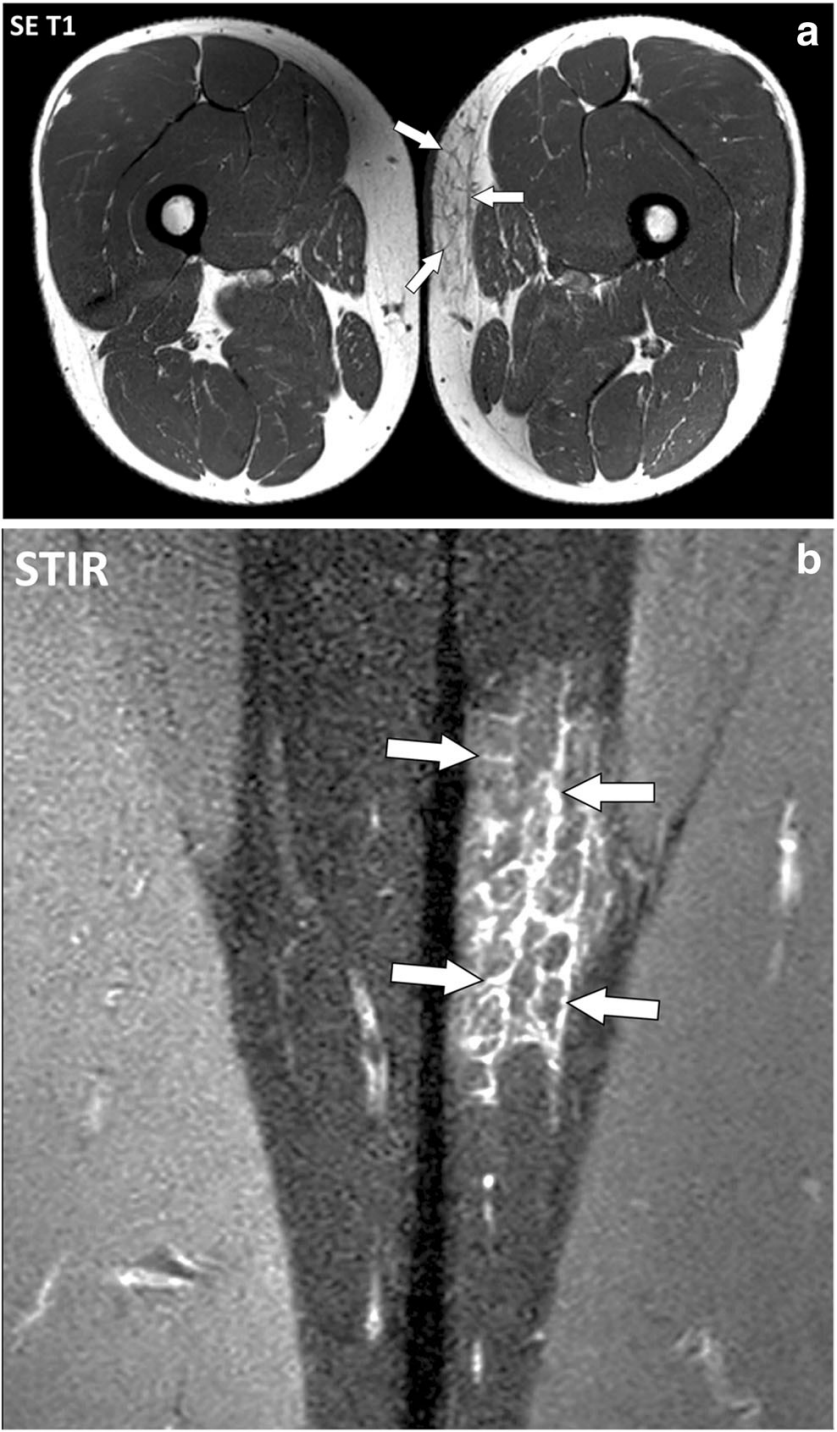
**a** Axial SE T1-weighted and (**b**) close-up coronal STIR images of the thighs of a 43-year-old male with dermatomyositis. The fascia superficialis of the left thigh (arrows) is thickened with fluid signal intensity

MR involvement of the fascia superficialis in active autoimmune diseases consists of thickening of the hypodermic reticular network of the fascia superficialis with fluid signal intensity (low signal on SE T1-weighted images and high signal on SE T2-weighted images) and enhancement after contrast material injection. Involvement of the fascia superficialis can extend deeply and involve the deep peripheral fascia and the connective tissue of the muscles. Fascia superficialis is a frequent target of dermatomyositis (Fig. 9).

### Deep fasciae

**Fig. 10:**
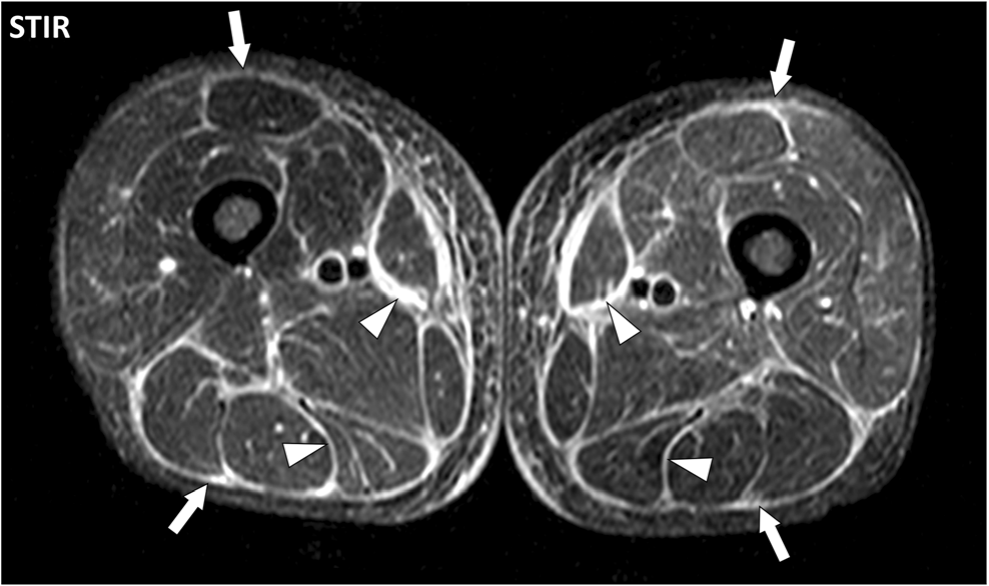
Axial STIR image of the thighs of a 50-year-old male with eosinophilic fasciitis. MRI demonstrates bilateral symmetrical diffuse involvement of the deep peripheral fasciae (arrows) and deep intermuscular fasciae (arrowheads), which are thickened with high signal intensity. The fascia superficialis is barely involved and the signal of the muscles is normal

**Fig. 11:**
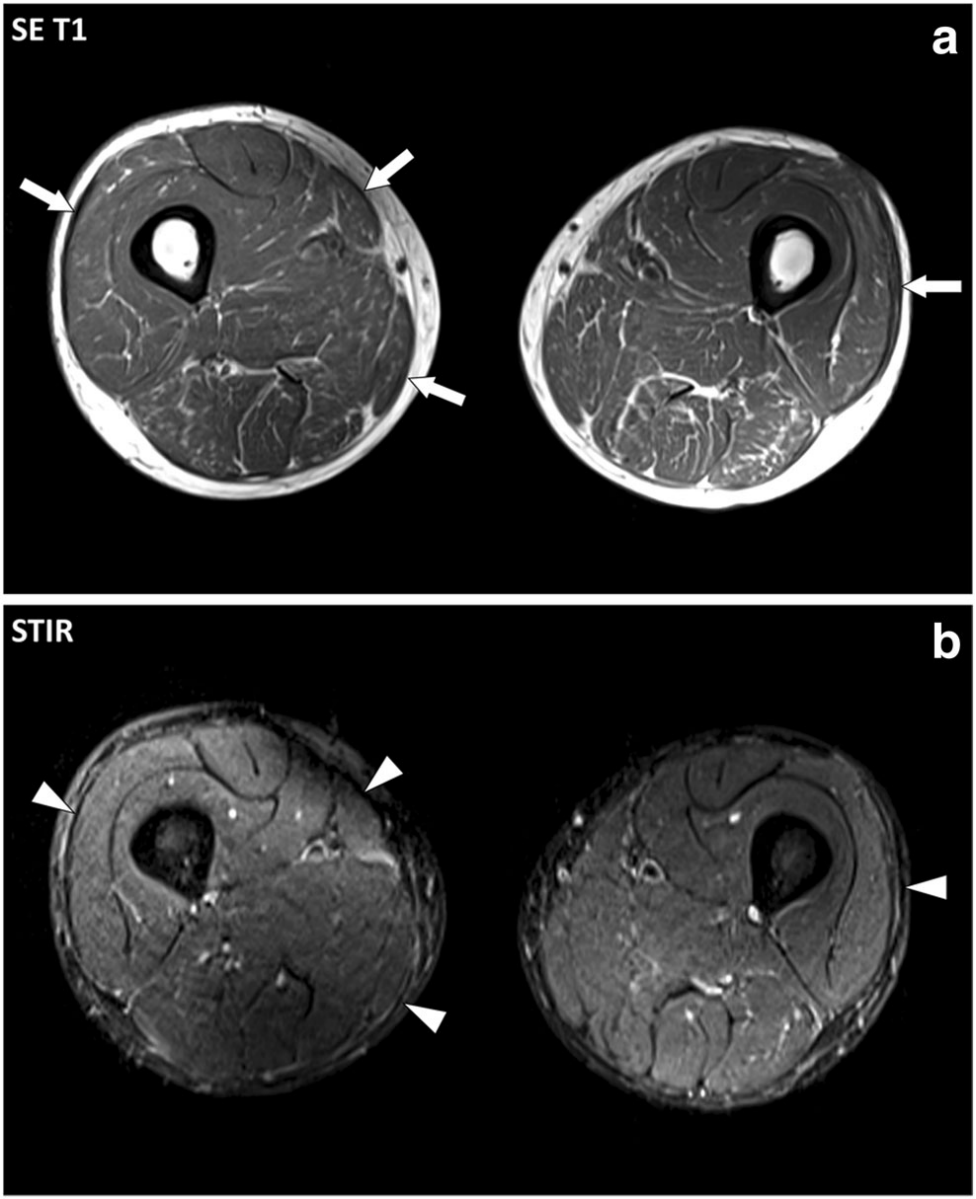
Axial (**a**) SE T1-weighted and (**b**) STIR images of the thighs of a 52-year-old male with eosinophilic fasciitis treated for a year. MRI demonstrates thickening (arrows) of the deep peripheral fasciae with low signal intensity on STIR images (arrowheads) suggesting fibrosis

**Fig. 12:**
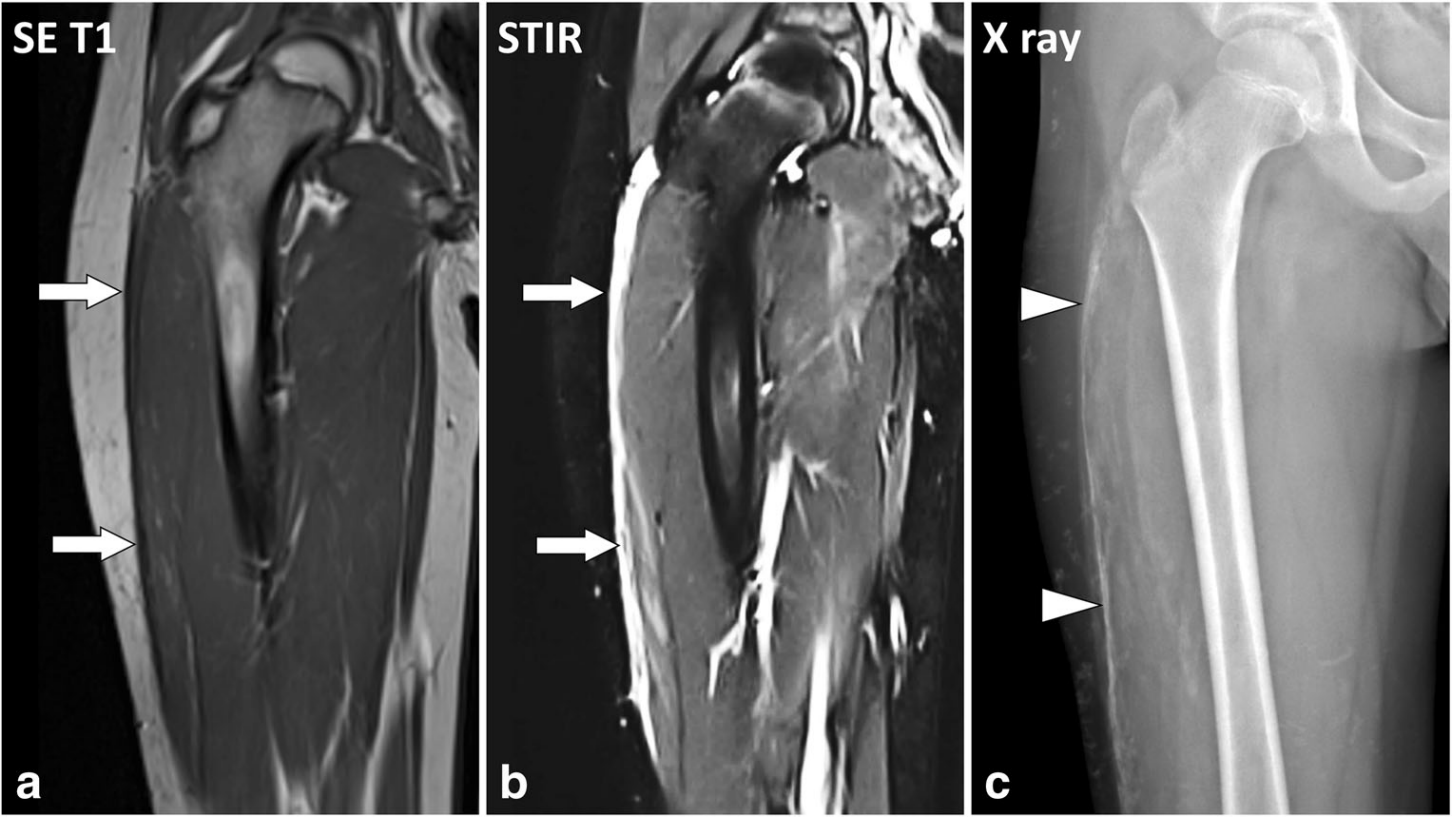
Coronal (**a**) SE T1-weighted and (**b**) STIR images and (**c**) plain X-ray of the right thigh of a 9-year-old boy with dermatomyositis. MRI shows thickening and fluid signal intensity of the deep peripheral fascia of the lateral part of the thigh (arrows). Plain X-ray demonstrates diffuse calcifications of the deep peripheral fascia (arrowheads), which are not visible at MRI

MR involvement of the deep fasciae in active autoimmune diseases consists of thickening of the deep peripheral fascia between hypodermis and muscles and/or the deep intermuscular fascia between muscles with fluid signal intensity and enhancement after contrast material injection. Deep fasciae are the main target of eosinophilic fasciitis (Shulman’s syndrome) (Fig. 10). Involvement of the deep fascia can extend to the adjacent structures and involve fascia superficialis, epi- and perimysium and/or periosteum [8]. Chronic involvement of the deep fasciae may induce persistent thickening of the fasciae with low signal intensity on T1- and T2-weighted images suggesting fibrosis (Fig. 11). Calcifications of the fasciae in autoimmune diseases are rare except for calcinosis in juvenile dermatomyositis and may be difficult to assess with MR (Fig. 12). Fluid collections are not part of the MR pattern of involvement of the deep fasciae in autoimmune diseases as opposed to necrotizing fasciitis [9].

### Retinacula

**Fig. 13:**
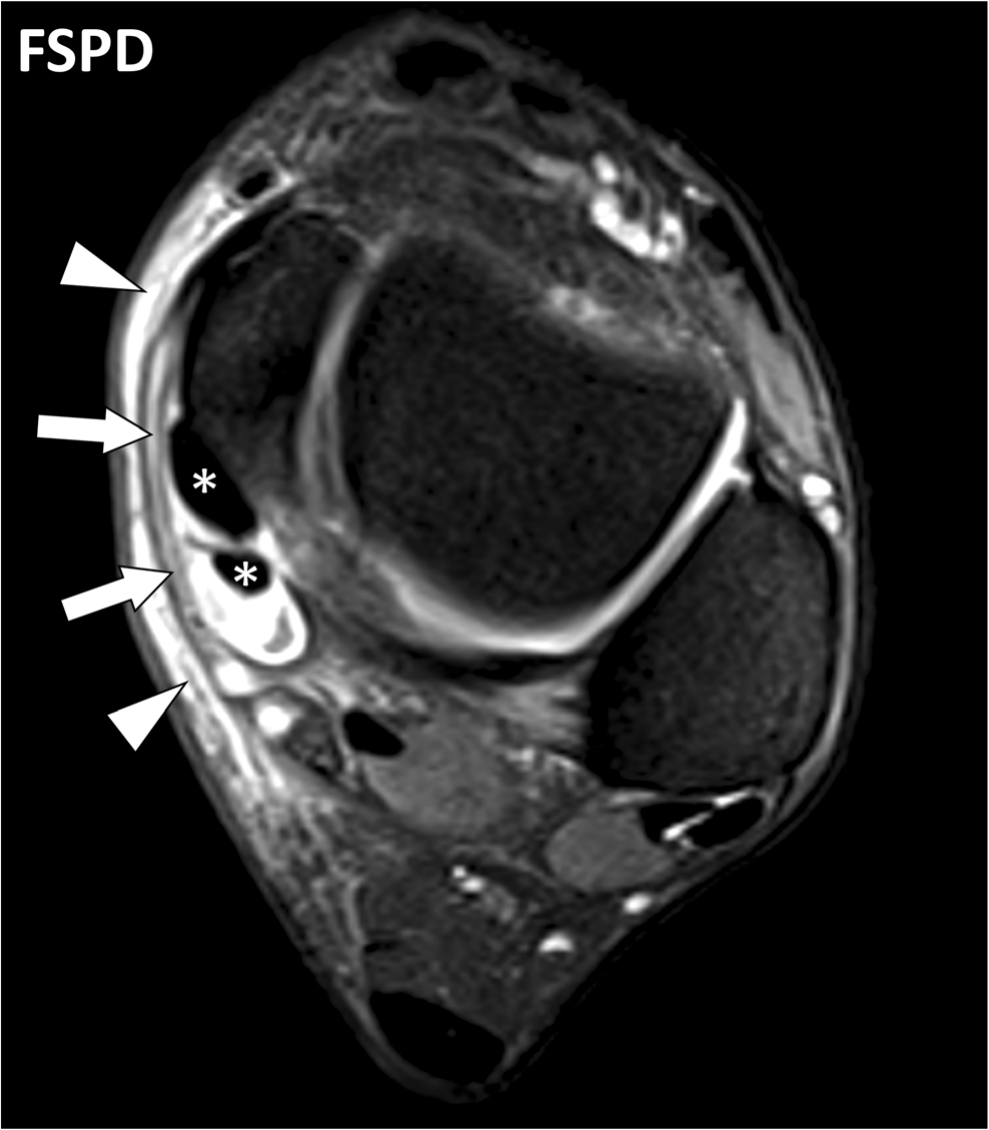
Axial FSPD-weighted image of the ankle of a 54-year-old male with psoriatic arthritis. Involvement of the flexor retinaculum (arrows) and subcutaneous fat (arrowheads) with thickening and fluid-like signal is associated with effusion of the synovial sheath (tenosynovitis) of the tibialis posterior and flexor digitorum longus tendons (asterisks)

**Fig. 14:**
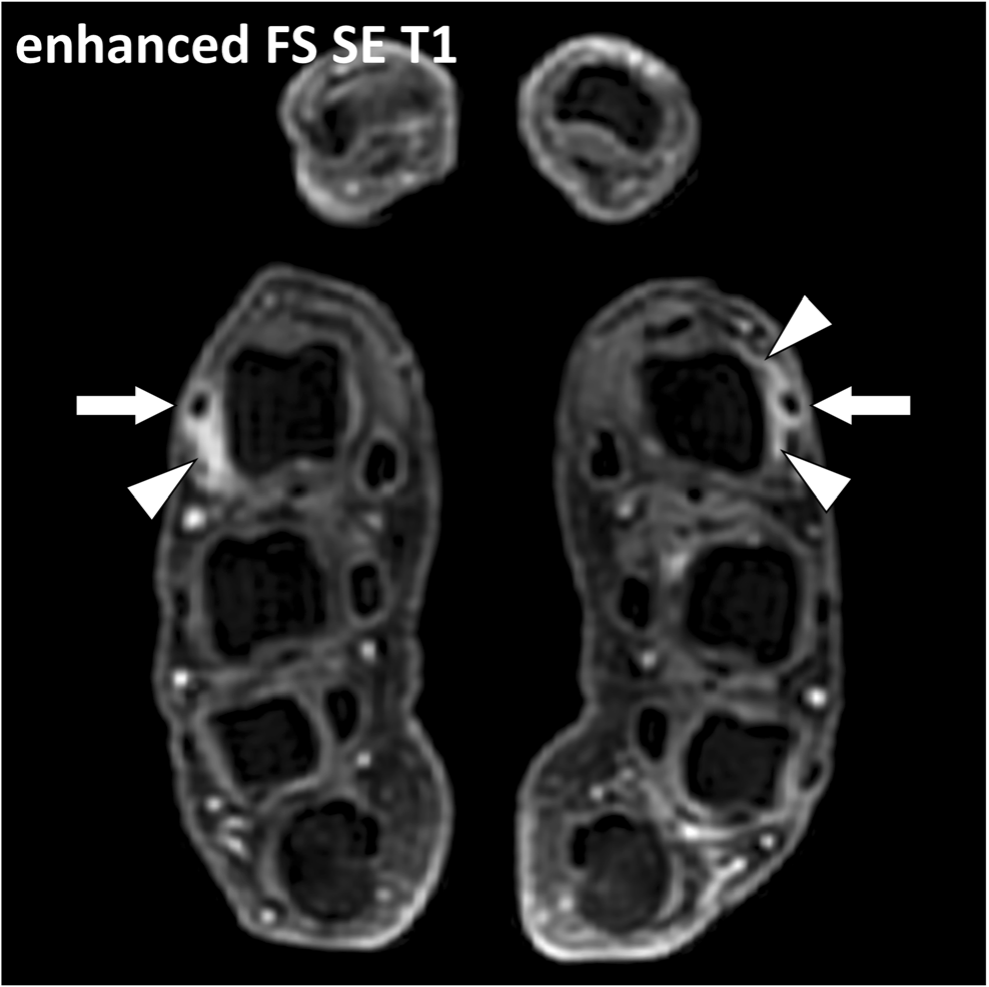
Contrast-enhanced fat-suppressed T1-weighted axial image of the hands of a 29-year-old female with seropositive rheumatoid arthritis. Peritendinitis of the extensor digitorum tendons of the second digits (arrows) is associated with thickening and enhancement of the connective tissue around the tendons and the dorsal parts of the sagittal bands (arrowheads). The term “peritendinitis” seems more appropriate than “tenosynovitis” as there is no synovial sheath around the extensor tendons at the level of the metacarpophalangeal joints

MR involvement of the retinacula in active autoimmune diseases consists of thickening of the retinacula with fluid signal intensity and enhancement after contrast material injection. It may be associated with tenosynovitis and involvement of the adjacent structures such as periosteum or subcutaneous tissues in seronegative arthritides (Fig. 13). In seropositive rheumatoid arthritis, involvement of the sagittal bands with peritendinitis of the extensor digitorum tendons is commonly observed (Fig. 14) [10].

### Epi- and perimysium

**Fig. 15:**
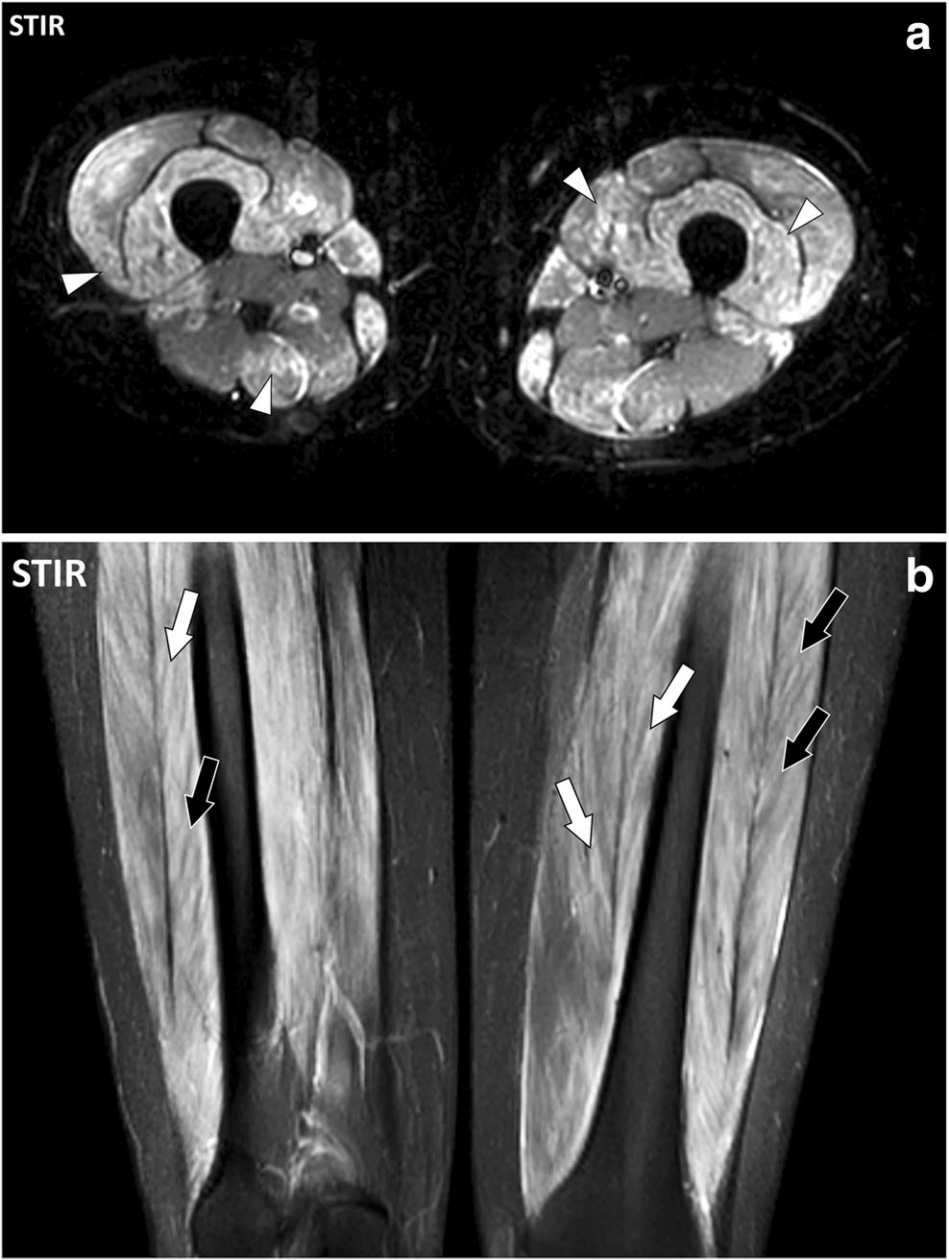
**a** Axial and (**b**) coronal STIR images of the thighs of a 16-year-old female with dermatomyositis and anti-TIF1-γ antibodies. MRI demonstrates bilateral symmetrical involvement of the muscles mostly in the quadriceps and gracilis muscles. Blood level of creatine phosphokinase (CPK) was normal. At MRI, involved muscles are heterogenous with a “salt and pepper” pattern in the axial plane (arrowheads) while they maintain a fibrillar organization with fluid signal intensity (white arrows) and low signal intensity (black arrows) striations in the coronal plane

**Fig. 16:**
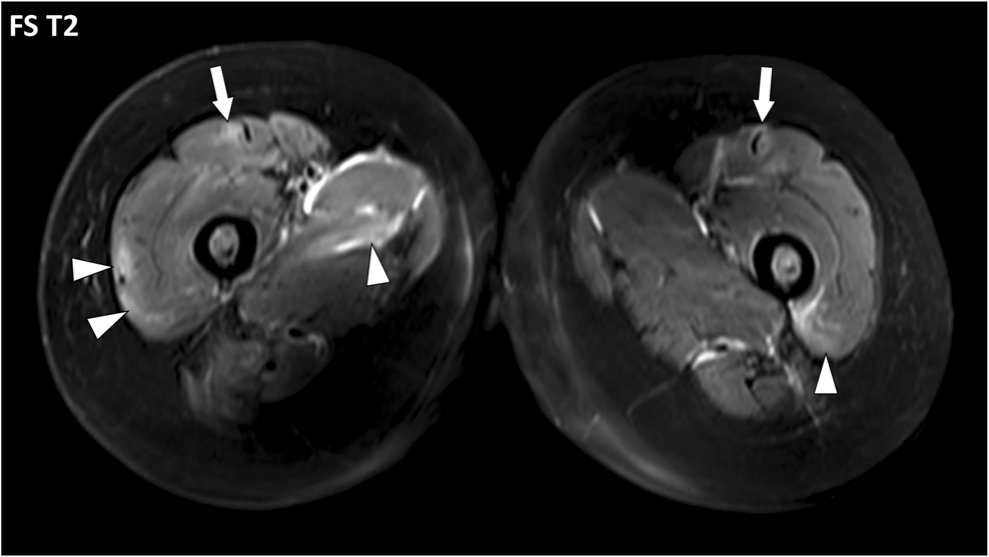
Axial fat-suppressed T2-weighted image of the thighs of a 49-year-old female with systemic lupus erythematosus, antiphospholipid syndrome and normal CPK. Multiple foci of increased signal intensity of the fascial system are depicted and they are all located at the periphery of the muscles near the deep fasciae (arrowheads) or near the fibro-muscular interfaces (arrows) suggesting involvement of the epimysium

MR involvement of the connective tissue of the muscles in active autoimmune diseases consists of ill-defined fluid signal intensity of the muscle tissues [11, 12]. At MRI, it may be impossible to distinguish involvement of the connective tissue from involvement of the muscle fibres (myositis) (Fig. 15). These changes may be focal or diffuse and may involve whole muscles or muscle groups (Fig. 16). Chronic inflammatory involvement may induce fatty transformation of the muscles with high signal intensity on T1- and T2-weighted images [13].

### Periosteum

**Fig. 17:**
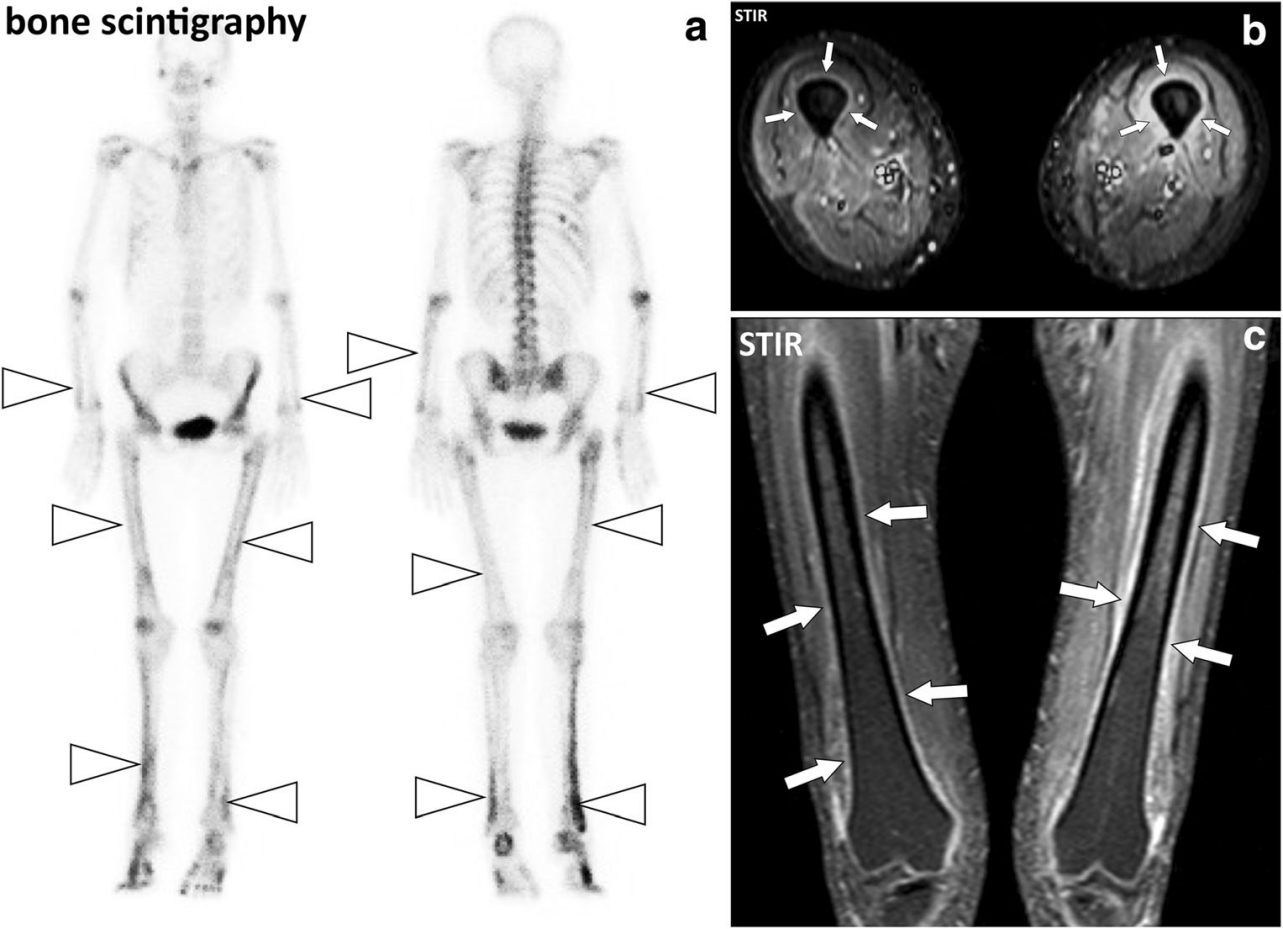
**a** Anterior and posterior whole-body bone scintigraphy, (**b**) axial and (**c**) coronal STIR images of the thighs of a 55-year-old female with pulmonary neoplasm and hypertrophic osteoarthropathy. Bone scintigraphy demonstrates symmetric linear increased uptake of tracer along the diaphysis and metaphysis of the long bones (arrowheads). MRI confirms periostitis with thickening and fluid signal intensity of the periosteum of the left femur and to a lesser degree of the right femur (arrows)

**Fig. 18:**
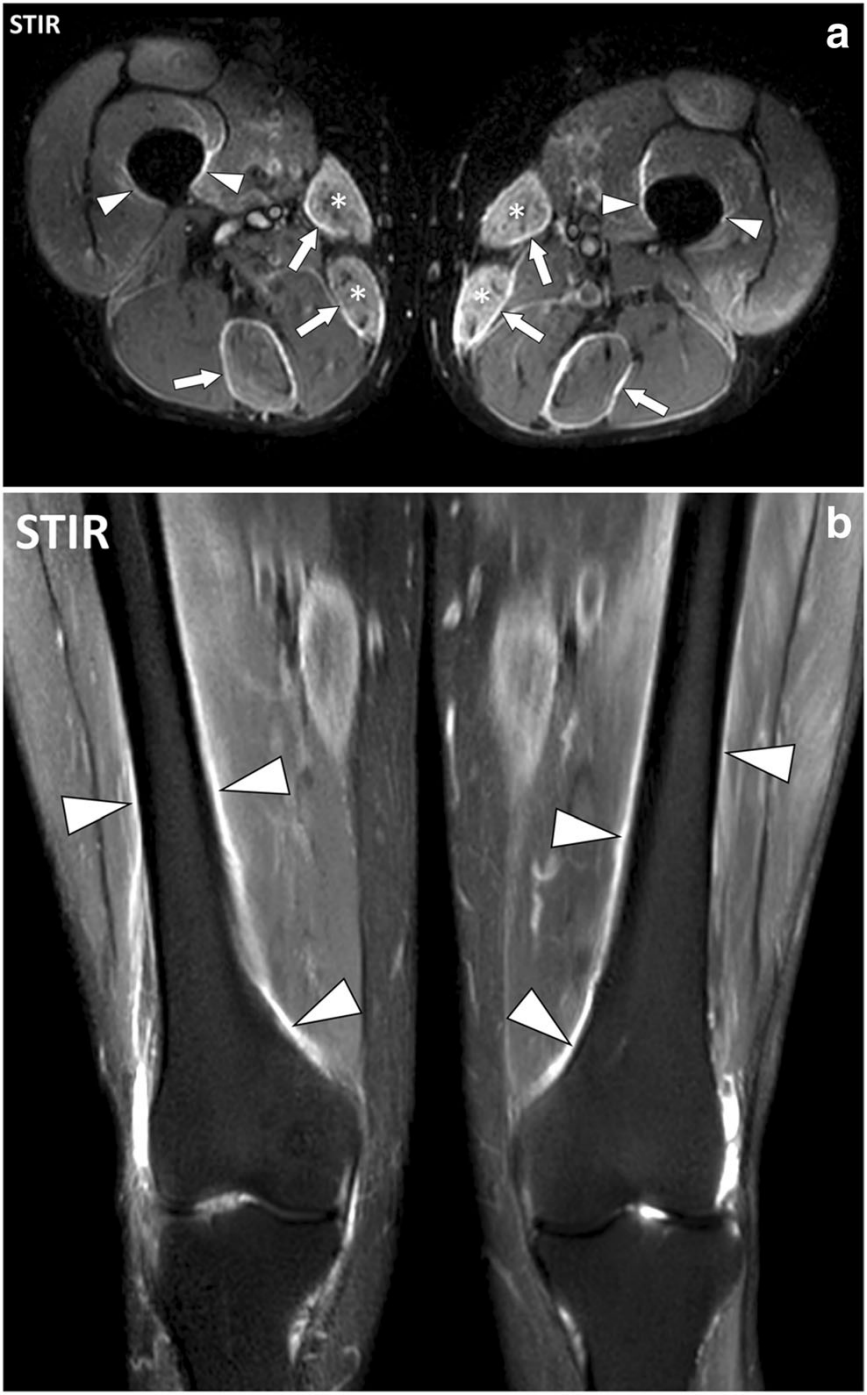
**a** Axial and (**b**) coronal STIR images of the thighs of a 51-year-old male with polymyositis. MRI demonstrates bilateral symmetrical involvement with myositis of the sartorius and gracilis muscles (asterisks), fasciitis of the deep fasciae adjacent to sartorius, gracilis and semitendinosus muscles (arrows) and periostitis mostly on the medial part of the diaphysis and metaphysis of the femurs (arrowheads)

MR involvement of the periosteum in active autoimmune diseases consist of thickening with fluid signal intensity and enhancement after contrast injection of the periosteum (periostitis). Periosteum can be the main target as in hypertrophic osteoarthropathy (Pierre-Marie-Bamberger syndrome) (Fig. 17) or accompany involvement of the deep fasciae or connective tissue of the muscles (Fig. 18).

### Holistic approach of autoimmune disorders of the musculoskeletal system

**Fig. 19:**
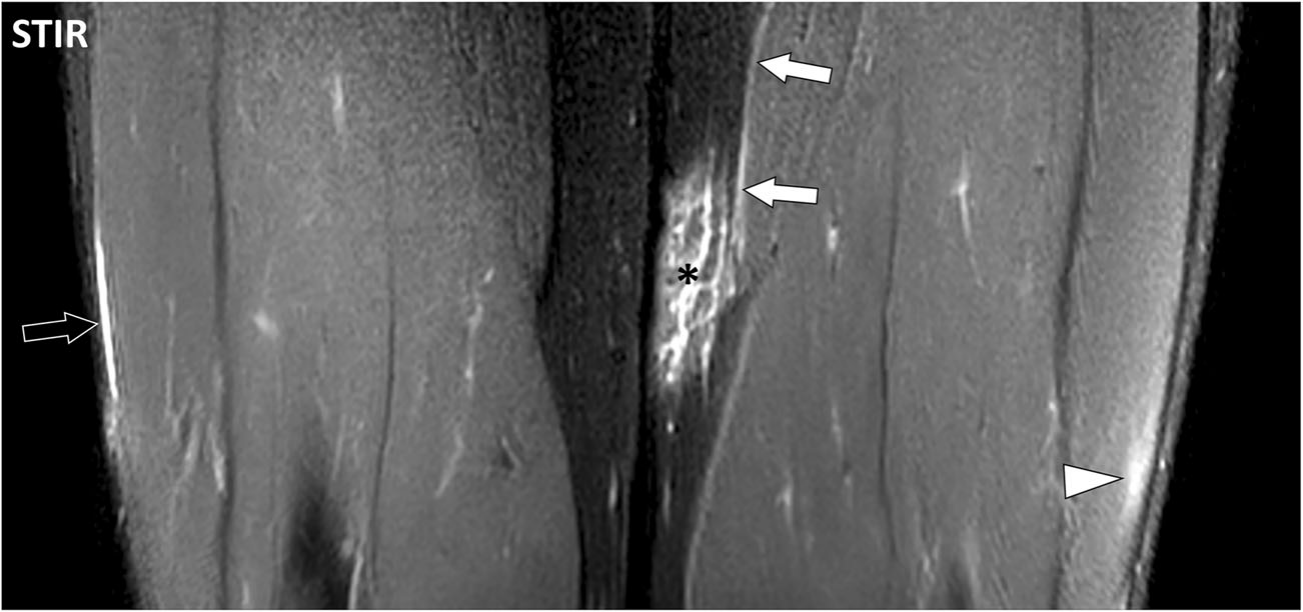
Coronal STIR image of the thighs of a 43-year-old male with dermatomyositis (same as in Fig. 9). Involvement of the fascia superficialis of the medial part of the left thigh (asterisk) extends to the adjacent deep peripheral fascia (white arrows). The deep peripheral fascia of the right thigh (black arrow) and the subaponeurotic connective tissues of the left vastus lateralis muscle (arrowhead) are also involved

Autoimmune inflammatory disorders may involve the fasciae as well as the other components of the connective skeleton (Fig. 19). There are however important variations among these conditions concerning the target tissue, intensity of the inflammation, type of cellular infiltrates, their natural history and, finally, their influence on the adjacent tissue such as the synovium, muscles or bones [14–19].

**Fig. 20:**
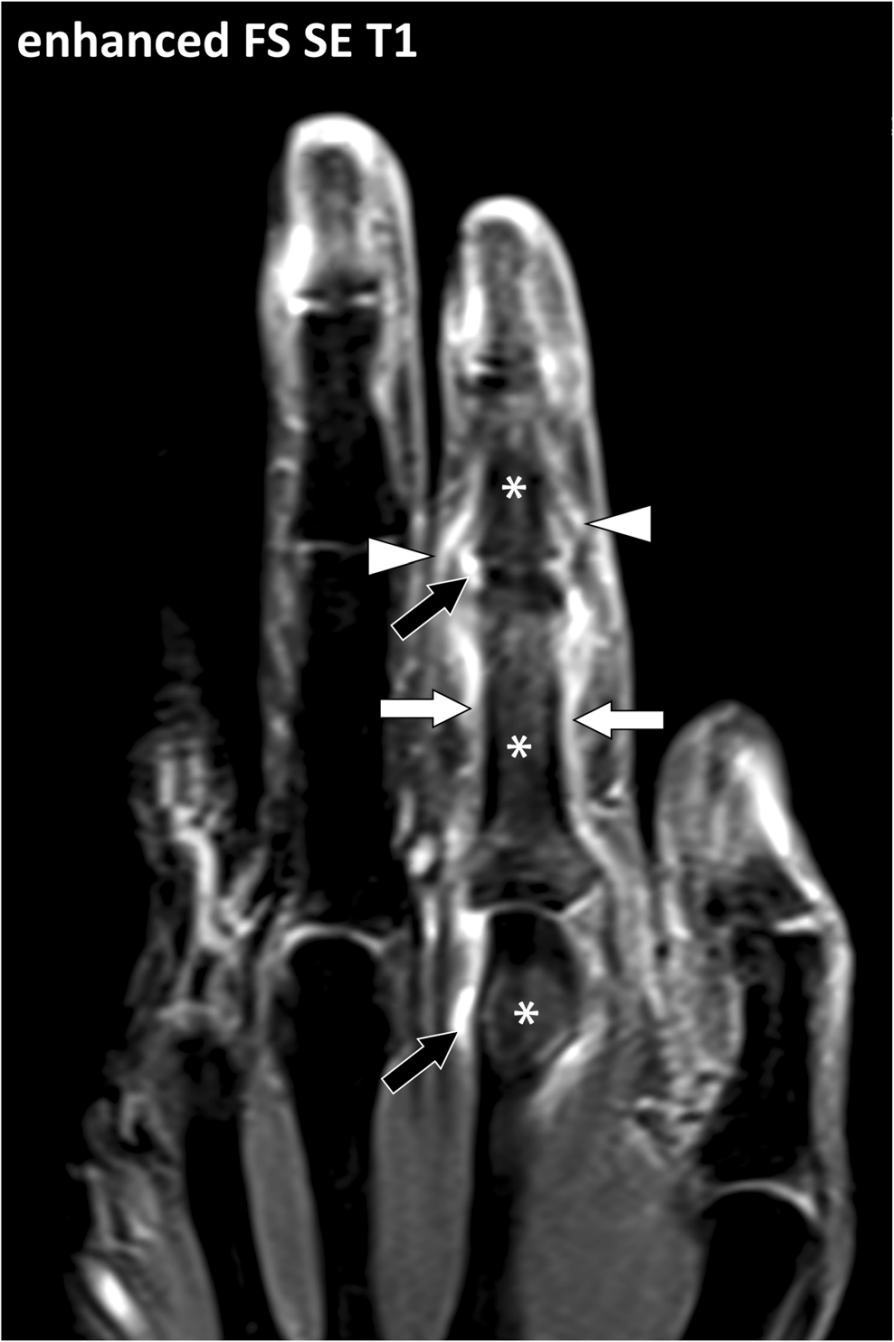
Contrast-enhanced fat-suppressed T1-weighted coronal image of the hand of a 35-year-old male with psoriatic arthritis and dactylitis of the second digit. MRI demonstrates periostitis of the proximal phalangeal bone with thickening and enhancement of the periosteum (white arrows) and osteitis (asterisks). These changes are associated with synovitis of the metacarpophalangeal and proximal interphalangeal joints (black arrows). The connective tissue located at the interfaces between tendons, joint capsules and bone is also involved (arrowheads)

**Fig. 21:**
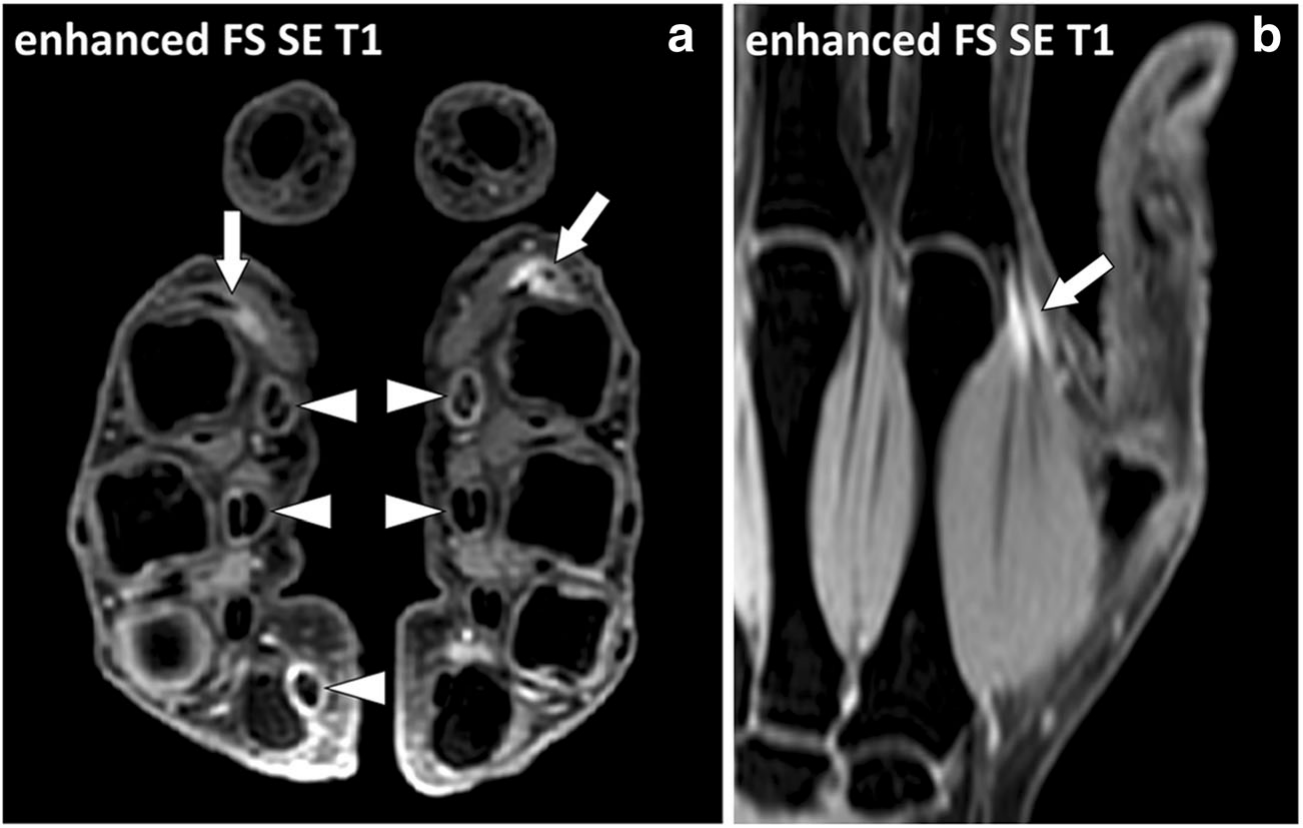
Contrast-enhanced fat-suppressed T1-weighted (**a**) axial image of the hands and (**b**) coronal reformat of the left hand of a 30-year-old female with seropositive non-erosive rheumatoid arthritis. MRI demonstrates inflammatory changes of the distal myo-tendinous junctions of the first dorsal interosseous muscles (arrows). Lack of synovitis suggests primary inflammatory involvement of the myo-tendinous junction. Tenosynovitis of the flexor digitorum tendons is also seen (arrowheads)

An overview of the most frequent rheumatological disorders with respect to fascial involvement can be summarized as follows. In seronegative arthritides, the connective tissue located at the interface between bones and tendons or capsules, i.e., entheses, is the target of the inflammatory reaction with predominant involvement of the axial skeleton and sacro-iliac joints in spondyloarthritis and predominant involvement of the extremities in psoriatic arthritis (Fig. 20). In seropositive arthritis, the connective tissue adjacent to the small synovial joints including the aponeuroses, retinacula and myo-tendinous junction (Fig. 21) are involved [20]. In polymyalgia rheumatica, the connective tissue adjacent to the large central joints of the limbs and the paraspinous areas are involved. In systemic lupus erythematosus as in systemic scleroderma, the connective tissue of the upper digestive tract and lungs are among the main targets but the retinacula, fasciae and capsule can be involved in a systemic or focal pattern [19].

It remains to be assessed whether the involvement of synovium, bone and muscles in these disorders results from primary synovial, bone and muscle diseases or is secondary to the inflammatory involvement of the fascial system [21].

## Conclusion

The fascial system is a complex network of connective tissue that interconnects all the components of the musculoskeletal system. The normal fascial system is relatively inconspicuous at MRI. MR patterns of fascial involvement in autoimmune diseases reflects the complex anatomy of the musculoskeletal fascial system.

## References

[1] Stecco C, Macchi V, Porzionato A, Duparc F, De Caro R (2011). The fascia: the forgotten structure. Ital J Anat Embryol.

[2] Adstrum S, Hedley G, Schleip R, Stecco C, Yucesoy CA (2017). Defining the fascial system. J Bodyw Mov Ther.

[3] Schleip R, Jäger H, Klingler W (2012). What is ‘fascia’? A review of different nomenclatures. J Bodyw Mov Ther.

[4] Kumka M, Bonar J (2012). Fascia: a morphological description and classification system based on a literature review. J Can Chiropr Assoc.

[5] Abu-Hijleh MF, Roshier AL, Al-Shboul Q, Dharap AS, Harris PF (2006). The membranous layer of superficial fascia: evidence for its widespread distribution in the body. Surg Radiol Anat.

[6] Benjamin M (2009). The fascia of the limbs and back – a review. J Anat.

[7] Herlin C, Chica-Rosa A, Subsol G (2015). Three-dimensional study of the skin/subcutaneous complex using in vivo whole body 3T MRI: review of the literature and confirmation of a generic pattern of organization. Surg Radiol Anat.

[8] Kirchgesner T, Dallaudière B, Omoumi P (2015). Eosinophilic fasciitis: typical abnormalities, variants and differential diagnosis of fasciae abnormalities using MR imaging. Diagn Interv Imaging.

[9] Malghem J, Lecouvet FE, Omoumi P, Maldague BE, Vande Berg BC (2013). Necrotizing fasciitis: contribution and limitations of diagnostic imaging. Joint Bone Spine.

[10] Nieuwenhuis WP, Krabben A, Stomp W (2015). Evaluation of magnetic resonance imaging-detected tenosynovitis in the hand and wrist in early arthritis. Arthritis Rheumatol.

[11] McMahon CJ, Wu JS, Eisenberg RL (2010). Muscle edema. AJR Am J Roentgenol.

[12] Smitaman E, Flores DV, Mejía Gómez C, Pathria MN (2018). MR imaging of Atraumatic muscle disorders. Radiographics.

[13] Dion E, Chérin P (2004). Use of muscular MRI in inflammatory myopathies. Rev Med Interne.

[14] Schanz S, Fierlbeck G, Ulmer A (2011). Localized scleroderma: MR findings and clinical features. Radiology.

[15] Boutry N, Hachulla E, Zanetti-Musielak C, Morel M, Demondion X, Cotten A (2007). Imaging features of musculoskeletal involvement in systemic sclerosis. Eur Radiol.

[16] Baumann F, Brühlmann P, Andreisek G, Michel BA, Marincek B, Weishaupt D (2005). MRI for diagnosis and monitoring of patients with eosinophilic fasciitis. AJR Am J Roentgenol.

[17] Moulton SJ, Kransdorf MJ, Ginsburg WW, Abril A, Persellin S (2005). Eosinophilic fasciitis: spectrum of MRI findings. AJR Am J Roentgenol.

[18] Maurer B, Walker UA (2015). Role of MRI in diagnosis and management of idiopathic inflammatory myopathies. Curr Rheumatol Rep.

[19] Schanz S, Henes J, Ulmer A (2013). Magnetic resonance imaging findings in patients with systemic scleroderma and musculoskeletal symptoms. Eur Radiol.

[20] Rowbotham EL, Freeston JE, Emery P, Grainger AJ (2016). The prevalence of tenosynovitis of the interosseous tendons of the hand in patients with rheumatoid arthritis. Eur Radiol.

[21] Noda K, Yoshida K, Ukichi T (2017). Myalgia in patients with Dermatomyositis and Polymyositis is attributable to fasciitis rather than Myositis: a retrospective study of 32 patients who underwent Histopathological examinations. J Rheumatol.

